# Antioxidant Activity of a Red Lentil Extract and Its Fractions

**DOI:** 10.3390/ijms10125513

**Published:** 2009-12-23

**Authors:** Ryszard Amarowicz, Isabell Estrella, Teresa Hernández, Montserrat Dueñas, Agnieszka Troszyńska, Kosińska Agnieszka, Ronald B. Pegg

**Affiliations:** 1 Division of Food Science, Institute of Animal Reproduction and Food Research of the Polish Academy of Sciences, Tuwima Street 10, 10-474 Olsztyn, Poland; E-Mails: a.troszynska@pan.olsztyn.pl (A.T.); a.kosinska@pan.olsztyn.pl (A.K.); 2 Instituto de Fermentationes Industriales (CSIC), Juan de la Cierva 3, 2800 Madrid, Spain; E-Mails: iestrella@ifi.csic.es (I.E.); thernandez@ifi.csic.es (T.H.); 3 Grupo de Investigación en Polifenoles, Unidad de Nutrición y Bromatología, Facultad de Farmacia, Universidad de Salamanca, 37007-Salamanca, Spain; E-Mail: mduenas@usal.es; 4 Department of Food Science and Technology, The University of Georgia, 100 Cedar Street, Athens, GA 30602-7610, USA; E-Mail: rpegg@uga.edu

**Keywords:** free radicals, red lentils, phenolic compounds, tannins, HPLC-ESI-MS

## Abstract

Phenolic compounds were extracted from red lentil seeds using 80% (v/v) aqueous acetone. The crude extract was applied to a Sephadex LH-20 column. Fraction 1, consisting of sugars and low-molecular-weight phenolics, was eluted from the column by ethanol. Fraction 2, consisting of tannins, was obtained using acetone-water (1:1; v/v) as the mobile phase. Phenolic compounds present in the crude extract and its fractions demonstrated antioxidant and antiradical activities as revealed from studies using a *β*-carotene-linoleate model system, the total antioxidant activity (TAA) method, the DPPH radical-scavenging activity assay, and a reducing power evaluation. Results of these assays showed the highest values when tannins (fraction 2) were tested. For instance, the TAA of the tannin fraction was 5.85 μmol Trolox^®^ eq./mg, whereas the crude extract and fraction 1 showed 0.68 and 0.33 μmol Trolox^®^ eq./mg, respectively. The content of total phenolics in fraction 2 was the highest (290 mg/g); the tannin content, determined using the vanillin method and expressed as absorbance units at 500 nm per 1 g, was 129. There were 24 compounds identified in the crude extract using an HPLC-ESI-MS method: quercetin diglycoside, catechin, digallate procyanidin, and *p*-hydroxybenzoic were the dominant phenolics in the extract.

## 1. Introduction

Phenolic compounds originating from edible and non-edible plant parts possess antioxidant activity. They display the capability to inhibit or delay the oxidation of lipids, proteins, and DNA by affecting the initiation or propagation of oxidizing chain reactions. Natural phenolic antioxidants can scavenge reactive oxygen and nitrogen species (RONS) thereby preventing the onset of oxidative diseases in the body [1,2]. A positive correlation between the consumption of phenolic-rich foods and a decrease of several chronic diseases has been shown to exist from epidemiological studies [3,4].

Leguminous seeds belong to plant foods which are generally rich in phenolic compounds, including condensed tannins. The antioxidant activity of phenolic compounds extracted from leguminous seeds has been investigated using several *in vitro* chemical assays [5–20]. Lentils are a leguminous seed that have high levels of natural antioxidants [21–27]. A number of researchers have confirmed the high antioxidant potential arising from tannin constituents present in plant extracts [28–38].

The objectives of this research were to investigate the antioxidant and antiradical activities of a crude acetone red lentil seed extract and its isolated low-molecular-weight phenolics and tannin fractions.

## 2. Results and Discussion

### 2.1. Content of Total Phenolics and Condensed Tannins

The content of total phenolics in fraction 1 was 3× lower than that determined in the crude extract. The highest amount (290 mg/g) was found in fraction 2 (Table 1). In fraction 1, the dominant compounds were sugars; these eluted from the column by ethanol together with low-molecular-weight phenolic compounds. The total phenolics content of the crude acetone red lentil extract, was higher than values reported for crude extracts of red bean (55 mg/g) [10] and pea (23 mg/g) [9]. A higher quantity was found, however, in the crude extracts of vetch (66 mg/g) [14], green lentil (68 mg/g) [27], and adzuki bean (90 mg/g) [11].

The marked content of total phenolics in the tannin fraction separated from the crude extract using Sephadex LH-20 column chromatography has been reported for other leguminous seeds (e.g., beach pea, faba bean, pea, vetch, adzuki, bean, green lentil) [9–11,14,31], canola hulls [30], and evening primrose [33].

The content of tannins, expressed as absorbance units at 500 nm per g, in fraction 2 (129) was nearly 2× greater than that determined in the crude extract (70). For the low-molecular-weight phenolics fraction, a vanillin-positive reaction was noted thereby suggesting that catechin/epicatechin and other flavan-3-ols had eluted from the Sephadex LH-20 column with ethanol [39]. In numerous papers, the presence of tannins has been reported in leguminous seeds [6,7,11,16,34,36,37].

### 2.2. UV Spectra

**Figure 1.**
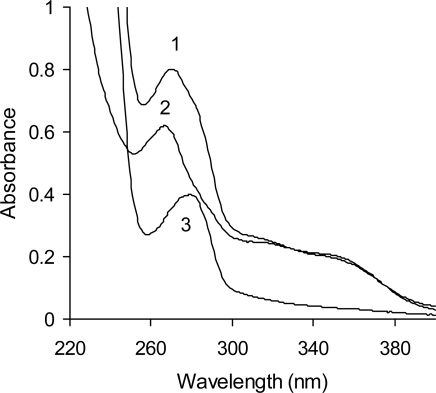
UV spectra of a red lentil acetonic extract and its fractions (1–extract; 2–fraction 1; and 3–fraction 2).

The UV maxima observed from the spectra of phenolic compounds present in the crude extract as well as fractions 1 and 2 (Figure 1, Table 1) were 271, 267, and 280 nm, respectively. The wavelength of 280 nm is typical for condensed tannins [9,10]. Absorption bands (shoulders) at wavelengths ranging between 320 and 350 nm in the spectra of the crude extract and fraction 1 confirm the presence of phenolic acids, flavanones, and/or flavanans [40].

### 2.3. Total Antioxidant Activity

The greatest value of the Total Antioxidant Activity was noted for the tannin fraction: 5.85 μmol Trolox^®^ eq./mg (Table 1). Much less active was the crude extract (0.68 μmol Trolox^®^ eq./mg) and fraction 1 (0.33 μmol Trolox^®^ eq./mg). An acetone extract of pea seed coats was characterized with a TAA value of 3.6 μmol Trolox^®^ eq./mg [15]. A vetch extract and its low-molecular-weight phenolics and tannin fractions exhibited TAA values of 0.79, 0.40, and 6.40 μmol Trolox^®^ eq./mg, respectively [14]. The extracts of leguminous seeds have been characterized by TAAs ranging from 0.30 (pea) to 1.76 μmol Trolox^®^ eq./mg (adzuki bean) [34].

### 2.4. Antioxidant Activity in the β-Carotene-linoleate Model System

**Figure 2.**
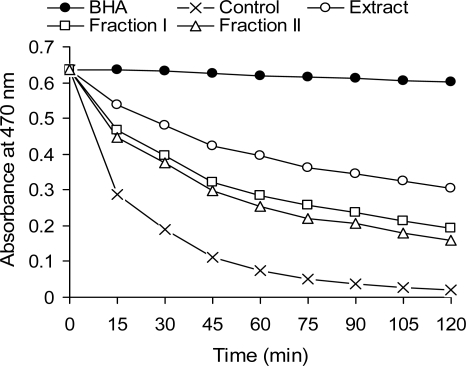
Antioxidant activity of a red lentil acetonic extract and its fractions in a *β*-carotene-linoleate model system, as measured by changes in absorbance at 470 nm.

The acetonic extract of phenolic compounds from the red lentil crude extract as well as fractions 1 and 2 exhibited varying degrees of antioxidant activity in the *β*-carotene-linoleate model system (Figure 2). The antioxidative effect of the samples on the coupled oxidation of linoleic acid and *β*-carotene was greatest for the crude extract, but it is important to remember that addition of the tannin fraction to the emulsion was only at a 1 mg level. On the other hand, the tannin fraction might be quickly oxidized under the assay conditions employed and therefore not as effective an antioxidant in the emulsion model. Using the same technique, similar or lower antioxidant activities were found for extracts of leguminous seeds from faba bean, broad bean, green lentil, and everlasting bean [12,27].

### 2.5. Reducing Power

**Figure 3.**
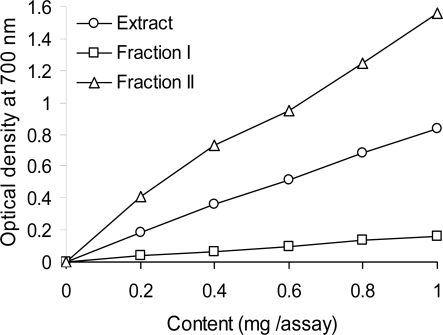
Reducing power of a red lentil acetonic extract end its fractions, as measured by changes in optical density at 700 nm.

Figure 3 displays the reducing power of the red lentil acetone extract and its fractions. Data indicates that fraction 2 exhibited a greater reducing power than either the crude extract or fraction 1. The reducing power of red lentil tannins was similar to that reported by Amarowicz *et al.* [31] for canola hull tannins. In cited studies, the reducing power of tannins recovered from beach pea, evening primrose, and faba bean extracts were more than 2× stronger than that of fraction 2 reported in this work. The same relation between the reducing power of the crude extract and its low-molecular-weight phenolics and tannin fractions were observed for vetch [14] and pea [9].

### 2.6. Scavenging of the DPPH Radical

**Figure 4.**
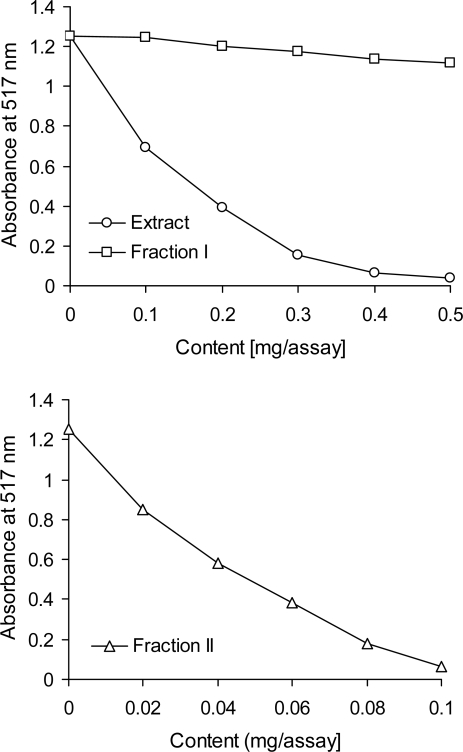
Scavenging effect of a red lentil acetonic extract and its fractions on the DPPH radical, as measured by changes in absorbance at 517 nm.

The strong antiradical scavenging effect of tannins separated from the red lentil acetonic extract was confirmed in the DPPH• assay (Figure 4). The tannin fraction exhibited an antiradical activity several times greater than that of the crude extract. The low-molecular-weight phenolics fraction (*i.e.*, fraction 1) was found to be a poor scavenger of DPPH•. The antiradical activity of the tannin fraction observed in this study is in accordance with literature data. The scavenging effects of condensed tannins from beach pea, canola hulls, evening primrose, and faba bean on the DPPH radical have been described by Amarowicz *et al.* [31]. Radical-scavenging activity of condensed tannin polymers from several plant sources against DPPH• has been discussed by Muir [38]. Shiriwardhana and Shahidi [41] reported that a whole almond seed extract scavenged 21% (at concentration at 100 ppm) and 73% (at 200 ppm) of the DPPH radical. In one study, a 100% scavenging activity of the DPPH radical was noted for brown almond skin and green shell extracts at 100 and 200 ppm concentrations, respectively.

### 2.7 Identification and Quantification of Phenolic Compounds

In Table 2 the wavelength of UV maximum, molecular ions, typical ion fragments, and the content of each compound separated and detected by the HPLC-PDA and HPLC-ESI-MS systems are given. Sinapic, *p*-hydroxybenzoic, *trans*-*p-*coumaric and *trans*-ferulic acids, gallic aldehyde, tryptophan, (+)-catechin, and (−)-epicatechin were identified by comparing their respective retention time and UV spectra with those of corresponding standards, and later confirmed by HPLC-ESI-MS.

Compounds with the same spectral shape and wavelength maximum (λ_max_ 278.9) have been characterized as flavanol monomers and procyanidin oligomers. Among these, compounds 8 and 13 from the HPLC-ESI-MS analysis exhibited a negative molecular ion [M-H]^−^ at an *m/z* of 451.1 corresponding to a flavanol monomer (either catechin or epicatechin) linked to glucose, and a fragment ion [F-H]^−^ at an *m/z* of 289 corresponding to either catechin or epicatechin. These compounds were identified as catechin glucoside and epicatechin glucoside, respectively, and the HPLC chromatogram agrees with the sequence of the elution time for the corresponding monomers. From the analysis by HPLC-ESI-MS, compounds 2, 11, and 19 showed a negative molecular ion [M-H]^−^ at an *m/z* of 577.1 corresponding to a procyanidin dimer, and a negative fragment ion [F-H]^−^ at an *m/z* of 289.1, which corresponds to either catechin or epicatechin. These peaks were identified as procyanidin dimers. Compounds 5 and 15 from the HPLC-ESI-MS analysis gave a negative molecular ion [M-H]^−^ at an *m/z* of 865.2 corresponding to a procyanidin trimer and a two fragments [F-H]^−^ at an *m/z* of 577 and 289.1 from a dimer and a monomer, respectively; these compounds have been identified as procyanidin trimers.

Compounds 3 and 6 exhibited a λ_max_ of 276.4 nm which corresponds to a prodelphinidin. In the HPLC-ESI-MS analysis, an [M-H]^−^ at an *m/z* of 593.1 from a dimer and two fragment ions [F-H]^−^ at an *m/z* of 289.1 and 305.1 were found and correspond to epi/catechin and galloepi/catechin. These compounds were classified as prodelphinidin dimers.

By HPLC-ESI-MS analysis, compound 4 showed a negative molecular ion [M-H]^−^ at an *m/z* of 881.3, which could correspond to a digallate procyanidin dimer, and two fragments [F-H]^−^ at an *m/z* of 577 and *m/z* 289 from a procyanidin dimer and a monomer (catechin or epicatechin). Compound 4 was identified as a digallate procyanidin dimer.

Compound 10 had a negative molecular ion [M-H]^−^ at an *m/z* of 745.1 and two fragments [F-H]^−^ at an *m/z* of 577 from a procyanidin dimer and 169.1 from gallic acid. Compound 10 was identified as procyanidin gallate.

Three quercetin glycosides (compounds 17, 22, and 23) were tentatively identified by their UV spectra. The quercetin diglycoside (compound 17) was identified because it presented a molecular ion [M-H]^−^ at an *m/z* of 625.3 corresponding to quercetin linked to a disaccharide (hexose + hexose), and a fragment ion [F-H]^−^ at an *m/z* of 301.2 from quercetin. Compounds 22 and 23 showed a UV spectrum similar to that of the quercetin glycoside, but possessed a second maximum with a hypsochromic shift to a lower wavelength and a shoulder at 267 nm, both corresponding to an acylated glycoside. Compounds 22 and 23 also had a molecular ion [M-H]^−^ at an *m/z* of 505.2 and a fragment ion [F-H]^−^ at an *m/z* of 301.4 from quercetin. Both peaks correspond to two different acylated quercetin hexoses.

A kaempferol derivative was also identified (compound 20) by UV spectra. This compound gave an [M-H]^−^ at an *m/z* of 447.1 and a fragment [F-H]^−^ at an *m/z* of 285, corresponding to kaempferol.

Compound 24 was identified by its UV spectrum as an apigenin derivative and confirmed by HPLC-ESI-MS analysis; it yielded an [M-H]^−^ at an *m/z* of 430.1 and a fragment [F-H]^−^ at an *m/z* of 269.1, corresponding to apigenin.

Compounds possessing a flavanol structure (monomers, oligomers, and gallates) were the most abundant (~50%) class of compounds detected in the red lentil acetonic extract. It is important to point out that prodelphinidins were in similar abundance (161.6 μg/g) as were the procyanidins (dimers and trimers comprising 161.5 μg/g). The monomers in free and glycosidic forms were plentiful (192.8 μg/g) in the red lentil acetonic extract. Flavonols and flavones were also detected in the samples with quercetin diglycoside being the most abundant for these flavonoid classes. Non-flavonoid compounds (e.g., phenolic acids) were scarce in this type of lentil, and represented a smaller percentage in the totality of the phenolic compounds (20%).

The obtained profile of individual phenolic compounds in the red lentil acetonic extract is in line with those previously reported for leguminous seeds. Catechin and epicatechin glucosides, quercetin glucoside, myricetin, and procyanidin B_1_ and B_3_ were the main phenolic compounds found in a crude extract of adzuki bean [11,36,37]. Glucosides of flavones and flavonols were determined in the cotyledon of peas (*Pisum sativum* L.) [42]. A high content of quercetin-3-*O*-glucoside and myricetin-3-*O*-glucoside was also determined in raw cowpeas (*Vigna sinensis* L.) [43]. Caffeic, *o*-coumaric, ferulic, and sinapic acids were determined in the crude extract of red bean [10], whereas vanillic, caffeic, *p*-coumaric, sinapic, and ferulic acids, as well as quercetin and kaempferol were found in a pea crude extract [9].

## 3. Experimental

### 3.1. Chemicals

All solvents used were of analytical grade unless otherwise specified. Methanol, acetone, hexanes, ethanol, acetonitrile, potassium ferricyanide, and trichloroacetic acetic were acquired from the P.O.Ch. Company (Gliwice, Poland). Butylated hydroxyanisole (BHA), *tert*-butylhydroquinone (TBHQ), *β*-carotene, linoleic acid, vanillin, Folin & Ciocalteu’s phenol reagent, polyoxyethylenesorbitan monopalmitate (Tween 40), Sephadex LH-20, and 2,2′-diphenyl-1-picrylhydrazyl radical (DPPH•) were obtained from Sigma (Pozna, Poland). Protocatechuic acid, protocatechuic aldehyde, *trans-p*-coumaric acid, (+)-catechin, (−)-epicatechin, dihydroquercetin, quercetin, quercetin-3-*O*-glucoside, quercetin-3-*O*-galactoside, quercetin-3-*O*-rutinoside, kaempferol-3-*O*-rutinoside, tryptophan, and 2′,4,4′,6′-tetrahydroxydihydrochalcone-2′-*O*-*β*-glucoside (phloridzin) were purchased from Extrasynthese (Genay Cedex, France).

### 3.2. Plant Material

Authenticated red lentil seeds from the 2008 harvest were obtained from the Plant Breeding Station in Olsztyn (Poland).

### 3.3. Extract Preparation

Red lentils were ground in a coffee mill and defatted with hexanes in a Soxhlet apparatus for 6–8 h. Phenolic compounds were then extracted from the raw material using 80% (v/v) acetone at a solids-to-solvent ratio of 1:10 (w/v) at 50 °C for 30 min [23]. Extraction was carried out in Erlenmeyer flasks using a shaking water bath (Elpan 357, Wrocław, Poland). The extraction was repeated 2×, supernates combined, and acetone evaporated under vacuum at 40 °C using a Büchi rotary evaporator. Residual water in the extract was removed by lyophilization. The prepared extract was stored at −20 °C until analyzed.

### 3.4. Column Chromatography

Separation of the crude acetone extract into its low-molecular-weight phenolics and tannin fractions was achieved according to the method described by Strumeyer and Malin [44]. A 2 g portion of the crude extract was suspended in 20 mL of 95% (v/v) ethanol and applied onto a chromatographic column (5 × 40 cm) packed with Sephadex LH-20 and equilibrated with 95% (v/v) ethanol. Low-molecular-weight phenolic compounds (fraction 1) were eluted from the column using ~1 L of 95% (v/v) ethanol. To collect the tannins (fraction 2) adsorbed on the column, a second mobile phase was employed; the column was treated with 500 mL of 50% (v/v) acetone. Organic solvents were evaporated from the fractions and water from the eluent of fraction 2 was removed by lyophilization.

### 3.5. Content of Total Phenolics

The content of total phenolic compounds in the crude extract and each fraction was estimated using Folin & Ciocalteu’s phenol reagent [32]. (+)-Catechin was used as a standard in this work.

### 3.6. Condensed Tannins

The content of condensed tannins in the crude extract and its fractions was determined using the modified vanillin assay [45]. Results were expressed as absorbance units at 500 nm per 1 g extract (A_500_/g).

### 3.7. UV Spectra

UV spectra of the extract and its fractions were recorded with a Beckman DU 7500 diode array spectrophotometer (Beckman Coulter, Inc., Brea, CA, USA).

### 3.8. Total Antioxidant Activity (TAA)

The determination of the TAA was carried out using a Randox kit (Randox Laboratories Ltd., Crumlin, UK) according to the procedure outlined by the supplier. A concentration of 2 mg extract or fraction/mL methanol was used in the assay. Results were expressed as μmol Trolox^®^ equivalents (eq.)/mg extract or fraction.

### 3.9. Antioxidant Activity in a β-Carotene-linoleate Model System

The antioxidant activity of the red lentil acetonic extract and its fractions was determined in an emulsion system using the method described by Miller [46]. Briefly, methanolic solutions (0.2 mL) containing 2 mg of crude extract or fraction 1, or 1 mg of fraction 2, were added to a series of tubes containing 5 mL of a prepared emulsion of linoleic acid and *β*-carotene stabilized with Tween 40. Immediately after addition of the emulsion to each tube, the zero-time absorbance reading at 470 nm was recorded. Samples were kept in a water bath at 50 °C and their absorbance values were recorded over a 120 min period at 15 min intervals.

### 3.10. Reducing Power

Reducing power of phenolics was determined as described by Oyaizu [30]. A suspension of the crude extract as well as fractions 1 and 2 in 1 mL of deionized water was mixed with 2.5 mL of 0.2 M phosphate buffer (pH 6.6) and 2.5 mL of 1% (w/v) potassium ferricyanide. The mixture was incubated at 50 °C for 20 min. Following this, 2.5 mL of 10% (w/v) trichloroacetic acid was added and the mixture was then centrifuged at 1750 × g for 10 min. A 2.5-mL aliquot of the upper layer was mixed with 2.5 mL of deionized water and 0.5 mL of 0.1% (w/v) FeCl_3_. The absorbance of the mixture was read at 700 nm with the spectrophotometer.

### 3.11. Scavenging of the DPPH Radical

The scavenging effect of phenolics from the red lentil acetonic extract and its two fractions was monitored as described by Amarowicz *et al*. [48]. A 0.1 mL methanolic solution containing between 0.5 and 2.5 mg of the crude extract or fraction 1 and between 0.02 and 0.1 mg of fraction 2 was mixed with 2 mL of deionized water and then added to a methanolic solution of DPPH• (1 mM, 0.25 mL). The mixture was vortexed for 1 min, left to stand at room temperature for 20 min, and absorbance of the solution was then read at 517 nm with the spectrophotometer.

### 3.12. HPLC-PAD Analysis

Lyophilized acetonic extract (150 mg) was dissolved in 2 mL of 80% (v/v) methanol and filtered through a 0.45 μm cellulose acetate filter (Millipore) before HPLC analysis. The chromatographic system was equipped with an autoinjector, a quaternary pump, a photodiode-array detector 2001 (Waters Corp., Milford, MA, USA), a Nova-Pak C_18_ column (300 × 3.9 mm; 4 μm), and Millenium software. The analytical conditions for separation are described by Dueñas *et al.* [49]. Two mobile phases were employed for elution: (A) water-acetic acid (98:2, v/v) and (B) water-acetonitrile-acetic acid (78:20:2, v/v/v). The gradient profile was 0 to 55 min, 100%–20% A; 55–70 min, 20%–10% A; 70–80 min, 10%–5% A; and 80–100 min, 100% B. The flow rate was 1 mL/min from the beginning to 55 min, and then increased to 1.2 mL/min until the 100 min point. The column was re-equilibrated between sample injections with 10 mL of acetonitrile and 25 mL of the initial mobile phase. Detection was performed by scanning from 210 to 400 nm at an acquisition speed of 1 s. A volume of 100 μL was injected per run. All samples were analyzed in duplicate.

### 3.13. HPLC-ESI-MS Analysis

After HPLC separation, mass spectra were obtained using a Hewlett Packard 1100 MSD (Palo Alto, CA, USA) equipped with an API source using an electrospray ionization (ESI) interface. The conditions of analysis have been reported by Dueñas *et al*. [49]. The solvent gradient and column used were the same as those for HPLC-PAD analysis. ESI conditions were as follows: negative-ion mode; nitrogen was employed as the nebulizing gas at 40 psi and drying gas at 10 L/min at 340 °C; voltage at the capillary entrance, 4,000 V; and variable fragmentation voltage, 100 V (*m/z* 200–1,000) and 250 V (*m/z* 1,000–2,500). Mass spectra were recorded from an *m/z* of 100 to 2,500.

### 3.14. Identification and Quantification of Phenolic Compounds

Chromatographic peaks were identified by comparing retention times, UV spectra, and HPLC-ESI-MS spectra with those of commercial standards (the standards employed are listed in the Materials section). Other compounds with UV spectra similar to those of hydroxycinnamates, flavanols (monomers and oligomers), gallates, flavonols, and dihydrochalcone were identified as derivatives of these compounds. Their chemical structures were confirmed by HPLC-ESI-MS.

Quantification was made by an external standard method according to the maximum of absorption for each compound. Calibration curves were prepared by injecting different volumes of standards from a stock solution over the concentration range observed for each compound. Calibration curves were then calculated as linear regressions of peak area *vs* concentration of the standard. The *trans-p*-coumaric acid derivative was quantified using the calibration curve of the corresponding free phenolic acid, (+)-catechin glucoside and (−)-epicatechin glucoside were expressed as (+)-catechin and (−)-epicatechin, respectively, while procyanidins and prodelphinidin were expressed using the calibration curve for (+)-catechin. Dihydrochalcone was expressed as phloridzin. The gallates were quantified as gallic acid and the quercetin glycosides were expressed as quercetin glucoside.

## 4. Conclusions

This paper describes the antioxidant and antiradical capacities of a red lentil acetone extract as well as its low-molecular-weight phenolics and tannin fractions. The tannin fraction showed the highest content of total phenolics; similar findings were found based on the tannin content, total antioxidant activity, antiradical activity, and reducing power. HPLC-PAD and HPLC-ESI-MS data revealed various classes of phenolic compounds present in the red lentil crude extract. Twenty four compounds were identified with quercetin diglycoside, catechin, digallate procyanidin, and *p*-hydroxybenzoic being the dominant phenolics present. Red lentil is a leguminous species that can provide an important daily source of phenolic compounds in human diets.

## Figures and Tables

**Table 1.** Total phenolics content, tannin content, total antioxidant activity, and UV spectral characteristics of a red lentil acetonic extract and its fractions.

**Analyzed material**	**Total phenolics (mg/g)**	**Tannins** (**A_500_/g**)	**Total Antioxidant Activity (μmol Trolox^®^eq./mg)**	***λ*_max_(nm)**
Crude extract	58 ± 2	70 ± 2	0.68 ± 0.03	271
Fraction 1	12 ± 1	1.52 ± 0.03	0.33 ± 0.02	267
Fraction 2	290 ± 8	129 ± 4	5.85 ± 0.20	280

**Table 2.** Phenolic compounds identified by HPLC-PAD and HPLC-ESI-MS in a red lentil acetonic extract.

**Compound number**	**λ_max_(nm)**	**[M-H]**^−^**(*m/z*)**	**Fragment Ions (*m/z*)**	**Compounds**	**Content (μg/g)**
1	302.5	153.1		Gallic aldehyde	13.45 ± 1.02
2	278.8	577.1	289	Procyanidin dimer (1)	20.68 ± 1.23
3	276.4	593.1	289.1	Prodelphinidin dimer (1)	154.8 ± 4.14
4	282.3	881.3	577; 289	Digallate procyanidin dimer	83.29 ± 3.69
5	278.8	865	577.1; 289	Procyanidin trimer (1)	48.2 ±2.11
6	276.5	593.1	289	Prodelphinidin dimer (2)	6.8 ± 0.19
7	255,5	137.1		*p*-Hydroxybenzoic acid	73.46 ± 2.09
8	278.8	451.1	289.1	Catechin glucoside	51.95 ±2.62
9	278.8	289		Catechin	36.02 ±1.36
10	277.2	745.1	577; 169	Procyanidin gallate	32.78 ±1.23
11	278.8	577	289	Procyanidin dimer (2)	18.7 ±1.14
12	277	203.1		Tryptophan	122.4 ±3.7
13	278.8	451.1	289	Epicatechin glucoside	6.65 ± 0.22
14	278.8	289		Epicatechin	98.21 ± 3.57
15	278.8	865	577.1; 289	Procyanidin trimer (2)	39.3 ±1.19
16	309	163		*trans*-*p*-Coumaric acid	38.84 ±2.11
17	256; 355.5	625.3	301	Quercetin diglycoside	287.84 ±14.3
18	322.9	193		*trans*-Ferulic acid	15.99 ±1.12
19	278.8	577	289	Procyanidin dimer (3)	26.6 ±1.28
20	265; 347.8	447,1	285	Kaempferol derivative	37.56 ±1.96
21	235.7	223.2		Sinapic acid	0.06 ± 0.02
22	256;	505.4	301.1	Quercetin hexose, acylated	21.17 ±1.03
23	267sh,	505.4	301.1	Quercetin hexose, acylated	0.27 ± 0.04
24	325, 335	430,1	269.1	apigenin hexose	14.45 ±1.08
